# When You Come to a Fork in the Road, Take It: Wnt Signaling Activates Multiple Pathways through the APC/Axin/GSK-3 Complex

**DOI:** 10.3390/cells12182256

**Published:** 2023-09-12

**Authors:** Chenchen Li, Emma E. Furth, Anil K. Rustgi, Peter S. Klein

**Affiliations:** 1 Division of Hematology-Oncology, Department of Medicine, Perelman School of Medicine, University of Pennsylvania, Philadelphia, PA 19104, USA; 2 Department of Pathology and Laboratory Medicine, Perelman School of Medicine, University of Pennsylvania, Philadelphia, PA 19104, USA; 3 Division of Digestive and Liver Diseases, Department of Medicine, Vagelos College of Physicians and Surgeons, Columbia University, 1130 St. Nicholas Avenue, New York, NY 10032, USA; 4 Herbert Irving Comprehensive Cancer Center, 1130 St. Nicholas Avenue, New York, NY 10032, USA

**Keywords:** Wnt, adenomatous polyposis coli (APC), glycogen synthase kinase 3 (GSK-3), Axin, beta-catenin, mTOR, cancer

## Abstract

The Wnt signaling pathway is a highly conserved regulator of metazoan development and stem cell maintenance. Activation of Wnt signaling is an early step in diverse malignancies. Work over the past four decades has defined a “canonical” Wnt pathway that is initiated by Wnt proteins, secreted glycoproteins that bind to a surface receptor complex and activate intracellular signal transduction by inhibiting a catalytic complex composed of the classical tumor suppressor Adenomatous Polyposis Coli (APC), Axin, and Glycogen Synthase Kinase-3 (GSK-3). The best characterized effector of this complex is β-catenin, which is stabilized by inhibition of GSK-3, allowing β-catenin entrance to the nucleus and activation of Wnt target gene transcription, leading to multiple cancers when inappropriately activated. However, canonical Wnt signaling through the APC/Axin/GSK-3 complex impinges on other effectors, independently of β-catenin, including the mechanistic Target of Rapamycin (mTOR), regulators of protein stability, mitotic spindle orientation, and Hippo signaling. This review focuses on these alternative effectors of the canonical Wnt pathway and how they may contribute to cancers.

## 1. Introduction

Glycogen synthase kinase-3 (GSK-3) is a ubiquitous protein kinase in eukaryotes, with orthologues in protozoans such as *Dictyostelium*, metazoans, and plants [1,2,3,4,5,6,7,8,9,10]. GSK-3 was first purified and named for its activity toward glycogen synthase [11,12], but multiple functions have since been defined. Distinctive features of GSK-3 include a propensity for processive phosphorylation at characteristically spaced serines and threonines, a preference to phosphorylate 4 residues N terminal to an already phosphorylated site, a tendency to suppress signaling pathways and promote protein degradation [13], a large array of disparate substrates, and sensitivity to lithium, which is observed in GSK-3 from cellular slime molds to humans [14,15]. GSK-3 has been studied extensively for its central role in common signaling pathways, including Wnt/β-catenin and receptor tyrosine kinase (RTK)/AKT signaling (notably insulin signaling), as well as roles in Hedgehog, Notch, and other pathways. Its potential role as a therapeutic target in bipolar disorder, neurodegenerative diseases, viral infections, and cancer has generated continued interest among both basic and biopharmaceutical scientists.

The primary purpose of this review is to discuss Wnt/GSK-3 signaling in cancer. Given space limitations and the availability of superb and detailed recent reviews on the broader aspects of GSK-3 in promoting cancer progression and on targeting GSK-3 in cancer, we refer the reader to those reviews [16,17,18] and the reviews cited above. Authoritative reviews of general aspects of the Wnt/β-catenin pathway are also available [19,20,21,22]. “Noncanonical Wnt” pathways that share the frizzled family of receptors but use distinct intracellular signaling mechanisms, for example, planar cell polarity and Wnt/calcium pathways, are also reviewed elsewhere [23,24,25,26]. Here, we briefly review the general features of GSK-3 and then focus on some less appreciated but important aspects of Wnt and GSK-3, especially on the mechanisms by which canonical Wnt signaling inhibits GSK-3 catalytic activity, novel mechanisms of β-catenin-independent signaling by the “canonical” Wnt pathway, and how these divergent pathways contribute to cancer pathogenesis.

## 2. General Features of GSK-3

### 2.1. GSK-3α and GSK-3β Are Closely Related Serine/Threonine Kinases

GSK-3 primarily phosphorylates serines and threonines. While it can phosphorylate tyrosine, this is mostly through autophosphorylation that occurs during translation [27]. GSK-3 falls within the CMGC (cyclin-dependent kinase (CDK), mitogen-activated protein kinase (MAPK), glycogen synthase kinase (GSK) and CDC-like kinase) family of kinases and has functional as well as sequence similarities with the dual specificity tyrosine phosphorylation-regulated kinases (DYRKs) [28]. In most vertebrates, GSK-3 proteins are encoded by two similar genes, *GSK3A* and *GSK3B* [29], that are mostly but not completely redundant. Birds lack *GSK3A* [30] and mice with homozygous deletions of *Gsk3a* are viable [31,32], indicating that *Gsk3b* can fulfill many of the essential functions of GSK-3. In contrast, the *Gsk3b* loss of function is either embryonic or perinatal lethal in mice, with different lethal phenotypes reported by different groups [33,34,35]. The ability to generate a nearly normal fetus with isolated tissue-specific defects (defects in palate or cardiovascular development) in mice lacking *Gsk3b* underscores the similar functions of these two closely related isoforms in non-affected tissues, although the reasons for the disparate phenotypes for *Gsk3b* knockouts has not been fully explained. Embryos with *Gsk3a/Gsk3b* double knockout (DKO) have not been observed despite extensive efforts to generate them; the loss of both genes is very likely an early lethal mutation, as DKO mouse embryonic stem cells (mESCs) have a severely limited capacity for differentiation [36].

### 2.2. Distinct Functions of GSK-3α and GSK-3β

Despite their largely overlapping functions, distinct functions for the two genes have emerged. The *Gsk3a* KO mice have behavioral and metabolic defects [31,32], reduced sperm motility [37], and shorter life span [38]. *GSK3A* was recently shown to suppress chromatin looping by modulating activity of the chromatin organizing complex cohesin, specifically through recruitment of the cohesin “unloader” WAPL, whereas *GSK3B* did not share this function [39]. *GSK3A* is also specifically required for the survival of AML cells [40]. This observation led to the development of small molecule inhibitors that preferentially inhibit one or the other isoform, an impressive achievement given the high similarity in the catalytic domains of the two proteins [41]. Selective inhibition of GSK-3α also reverses Fragile X disease phenotypes in mice [42]. GSK-3α and Gsk-3β phosphorylate mostly similar substrates but do have distinct substrate preferences in some cases [43,44]. Similarly, proximity labeling studies show that while the range of proteins that interact with GSK-3α and Gsk-3β are mostly similar, there are interesting differences between the two isoforms that were confirmed in independent studies [45,46].

### 2.3. A Multitude of GSK-3 Targets

GSK-3 was first identified as the third of five protein kinases that phosphorylate glycogen synthase (GS) [11,12,47], which incorporates UDP-glucose into glycogen. GSK-3 inhibits GS by phosphorylating the enzyme at an array of serines and threonines with characteristic spacing, and phosphorylation of these residues by GSK-3 depends on a preceding phosphorylation at the +4 position [48,49,50,51,52], organized in the SXXXSXXXSXXXS motif, where S represents serine or threonine and the most C-terminal position is phosphorylated by another, priming kinase; for GS, the priming kinase is casein kinase II, but this varies for other substrates. GSK-3 then moves in a processive manner in the N terminal direction. This pattern is also observed for β-catenin and for a subset of other GSK-3 substrates [1,5,53]. However, GSK-3 phosphorylates a broad range of substrates, many of which do not contain +4 priming sites, including phosphatase Inhibitor-2 (I-2), cyclin D1 [54], PTB-associated splicing factor (PSF) [55], and Tau protein [1,5,53], and the mechanisms of substrate recognition in these cases are less well characterized.

Rigorous proof that a substrate is a direct in vivo target of GSK-3 is difficult to obtain and lacking in most cases. Evidence from *GSK3* KO or knockdown combined with pharmacologic inhibition can demonstrate that a phosphorylation is dependent on GSK-3 but does not distinguish direct from indirect phosphorylation. In vitro kinase reactions with recombinant GSK-3 show that GSK-3 can phosphorylate the target, but do not address whether this occurs under physiological conditions. The presence of the SXXXS motif and evidence that phosphorylation depends on a priming step further supports GSK-3 as the relevant kinase, but, as above, not all GSK-3 substrates require priming, and priming phosphorylation is not an exclusive feature of GSK-3.

### 2.4. GSK-3 Frequently Functions within a Scaffold Complex

GSK3 frequently functions as part of a scaffold and supports scaffold assembly or stability. The Axin binding of GSK-3 is integral to the Wnt pathway, as it facilitates interaction of GSK-3 with the Adenomatous Polyposis Coli (APC) protein and with β-catenin, and at the same time shields the “Wnt signaling” pool of GSK-3 from regulation by RTK/AKT pathways [56]. The A kinase anchoring proteins (AKAP)-11 and AKAP220 bind GSK-3 and PKA [45,46,57]. The assembly of the AKT and PP2A on the β arrestin-2 scaffold is enhanced by GSK-3, which also binds to this complex and promotes PP2A-depedent inactivation of AKT [58,59]. Hence, inhibition of GSK-3, for example, by lithium, disrupts the β arrestin-2 complex, releasing AKT and PP2A and preventing the dephosphorylation and inactivation of AKT. This mechanism may explain why lithium enhances AKT phosphorylation and activity [58,59].

### 2.5. GSK-3 Is Constitutively Active and Is Inhibited by Diverse Signals

GSK-3 is unusual in being a constitutively active kinase that typically represses downstream signaling. The best characterized pathways that engage GSK-3 are the canonical Wnt pathway and RTK/AKT signaling. These pathways inhibit GSK-3 to activate effectors downstream of GSK-3. The classical example is insulin signaling, which inhibits GSK-3 by inducing phosphorylation of GSK-3α at serine-21 and Gsk-3β at serine-9, creating a pseudosubstrate that binds the active site and inhibits activity toward primed substrates [51,60]. Insulin thus activates GS by inhibiting GSK-3. Wnt signaling also inhibits GSK-3, but through a distinct mechanism that does not involve N terminal phosphorylation. Indeed, GSK-3 is sequestered in separate pools that isolate its functions, preventing crosstalk between Wnt and RTK signaling pathways.

### 2.6. GSK-3 Has Multiple Functions in Addition to Its Roles in Wnt and RTK Signaling

While this review focuses on GSK-3 in the Wnt pathway, it should be kept in mind that GSK-3 has multiple functions. GSK-3 also antagonizes Hedgehog and Notch signaling; these ligands also inhibit GSK-3 to activate downstream signaling. The roles of GSK-3 in RTK, Hedgehog, and Notch signaling are not discussed here given space constraints (see references above). Given its multiple functions, GSK-3 can play both tumor suppressive and tumor promoting roles in cancer, as thoroughly reviewed in [16]. Indeed, multiple GSK-3 inhibitors are currently being explored as single-agent and adjunctive cancer therapies [17].

## 3. Wnt Signaling

Wnt signaling directs early patterning of metazoan embryos, cell fate specification, tissue and cell polarity, somatic stem cell maintenance, and other functions [19,20,21,22,61]. Aberrantly activated Wnt signaling through genetic loss of Wnt suppressors drives diverse malignancies [62]. Elucidation of the Wnt pathway arose from a convergence of studies of mammalian cancer and the study of embryonic patterning in model organisms such as *Drosophila melanogaster*, *Xenopus laevis*, zebrafish, *Caenorhabditis elegans*, and mouse [21,61,63,64,65]. The founding members of the Wnt gene family were originally named *Int1*, a gene that induces neoplastic transformation in cultured breast epithelial cells when activated by proviral integration [66], and *Wingless* (*Wg*), a classical segment polarity gene in *Drosophila* [67,68,69,70,71,72]. Given their similar primary sequences and activities [69,72], the gene family (and signaling pathway) was christened “Wnt” for Wingless + Int [10,73].

The “canonical Wnt pathway” or the “Wnt/β-catenin pathway” refers to a broadly conserved pathway initiated by secreted Wnt proteins that signal through a conserved surface receptor/co-receptor complex and the intracellular Axin complex to inhibit GSK-3 and promote β-catenin-dependent activation of Wnt target genes. However, β-catenin is just one of several effectors of this pathway. We first present an outline of the Wnt/β-catenin pathway and the mechanisms that mediate transduction of an extracellular Wnt ligand to target gene activation. As the pathway through β-catenin has been amply reviewed in depth elsewhere, we then focus on GSK-3, addressing mechanisms that regulate GSK-3 activity and discussing effectors of GSK-3 regulated specifically by the Wnt signaling apparatus, as these topics tend to receive less attention in the many reviews on Wnt signaling.

### 3.1. Wnt/β Catenin Pathway

**Figure 1:**
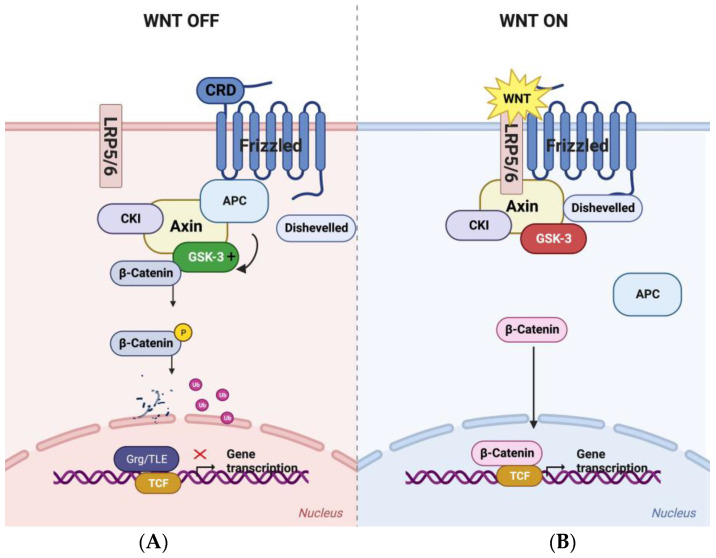
The canonical Wnt/β-catenin signaling pathway. (**A**) In the absence of Wnt signaling, an intracellular complex organized around the scaffold Axin binds APC, CK1α, GSK-3, and β-catenin. CK1α phosphorylates β-catenin at a “priming” site that then engages GSK-3, which phosphorylates a series of adjacent serines and threonines. Phosphorylated β-catenin is then ubiquitylated and degraded by the proteosome. As a result, Wnt target genes are repressed by a nuclear complex that includes TCF transcription factors and Grg/TLE co-repressors. (**B**) Wnt protein binding to the cysteine rich domain (CRD) of Frizzled proteins (7-pass integral membrane receptors) and LRP5/6 co-receptors (single pass) triggers phosphorylation of the C terminal domain of LRP5/6 by GSK-3 and CK1. Phosphorylated LRP5/6 recruits the Axin complex to the inner face of the cell membrane. At the same time, APC dissociates from the complex. These early steps in signaling cause GSK-3 inhibition and β-catenin stabilization. Accumulated β-catenin enters the nucleus, displaces the Grg/TLE co-repressor, and activates Wnt target gene transcription. (Diagrams made with BioRender).

In the absence of a Wnt ligand, the cell expends considerable effort to turn off the Wnt/β-catenin pathway (Figure 1A). An intracellular complex comprising the scaffold protein Axin, the classical tumor suppressor APC, and the protein kinases Casein Kinase 1α (CK1α) and GSK-3 maintains continuous phosphorylation of β-catenin at a bank of N-terminal serines and threonines with the characteristic SXXXS spacing. Axin directly binds each of these components through conserved domains, placing β-catenin in proximity to CK1α and GSK-3. CK1α phosphorylates β-catenin at a priming site (serine-45), allowing subsequent phosphorylation by GSK-3 at ser33, ser37, and thr41 [74]. APC also binds β-catenin directly through domains referred to as the 15 and 20 amino acid repeats in the central region of APC [75,76,77,78] (as cited by [79]). The affinity of these domains for β-catenin is increased by GSK-3- and CK1-dependent phosphorylation, and the interaction of β-catenin with these sites likely plays an important role in β-catenin phosphorylation within the Axin complex (see [79]). APC enhances the catalytic activity of GSK-3 and is required for maximal β-catenin phosphorylation [80,81,82]. The half-life of phosphorylated β-catenin is short, as newly synthesized protein is continually phosphorylated and degraded by the proteosome. Under these basal conditions, Wnt target genes are bound at specific sites by TCF family transcription factors associated with the Groucho (GRG)/Transducin-like enhancer of split (TLE) co-repressor complexes [83]. Wnt target genes are therefore actively repressed in the absence of Wnt signaling.

Wnt binding to the surface receptor Frizzled (FZD) and recruitment of the low-density lipoprotein receptor-related protein (LRP)-5 or -6 (LRP5/6) initiates signaling (Figure 1B). The first catalytic step is phosphorylation of the LRP5/6 C-terminus by GSK-3 and CK1α [84,85]. This series of phosphorylations recruits the Axin complex to LRP5/6 on the intracellular surface of the plasma membrane. GSK-3 plays an activating role at this step, in contrast to its more widely appreciated role as an Wnt antagonist within the Axin complex. Indeed, recruitment of the Axin complex to the surface leads to dissociation of APC, inhibition of GSK-3, and stabilization of β-catenin, which then accumulates, enters the nucleus, displaces the Groucho co-repressor complex, and activates transcription of Wnt target genes. Hence, GSK-3 promotes the initial intracellular steps in Wnt signaling in response to the ligand but antagonizes downstream signaling in the absence of the ligand. This dynamic regulation of β-catenin in the response to signaling means that total β-catenin increases as phosphorylated β-catenin is reduced; assessing the kinetics of β-catenin phosphorylation by measuring phosphorylated β-catenin relative to total β-catenin over time confirmed that the Wnt/β-catenin pathway is indeed regulated through inhibition of the catalytic activity of GSK-3 within the Axin complex [86].

The net effect of Wnt pathway activation is therefore to inhibit GSK-3. Wnt activation can be mimicked genetically by loss of function mutations in *APC*, *Axin*, or *GSK3*. These mutations, as well as mutation of the GSK-3 phosphorylation sites in β-catenin, activate Wnt/β-catenin target gene transcription. However, because of the redundancy of *Gsk3a* and *Gsk3b*, at least three of the four alleles must be knocked out to stimulate downstream signaling, and maximal activation is only seen when all four loci are disrupted [36]. Similarly, direct GSK-3 inhibitors such as lithium [14], inhibitory peptides like the heat-shock-factor-1-derived L807mts [87] and the GSK-3 interaction domain (GID) of Axin [88], or small molecule GSK-3 inhibitors stabilize β-catenin and activate canonical Wnt signaling. Inhibitors such as CHIR99021 are commonly used as “Wnt activators”, but it should be noted that GSK-3 inhibitors also affect Wnt-independent functions of GSK-3, including GSK-3 functions downstream of RTK/AKT signaling.

Several mechanisms have been put forward to explain how Wnts inhibit GSK-3 activity, and it is likely that each of these contribute to Wnt signaling. We and others showed that APC directly enhances the catalytic activity of GSK-3 [80,82] and that Wnt signaling promotes the dissociation of APC from the Axin complex within minutes of Wnt binding to the surface receptor [80,89,90,91] (although APC dissociation was not observed in an in vivo study using bimolecular fluorescence complementation to visualize Axin complex dynamics in *Drosophila* [92] or in an examination of the complex in HEK293T cells using native gel electrophoretic methods [93]). Thus, in our model, the dissociation of APC reduces GSK-3 activity toward β-catenin, allowing accumulation of unphosphorylated β-catenin. This model is consistent with the observation that oncogenic mutations in *APC,* which prevent its interaction with Axin, also impair β-catenin phosphorylation, mimicking Wnt-dependent activation. The model also predicts that other effectors of Axin-bound GSK-3 should be activated by Wnt signaling, as described in greater detail below [80,94]. An alternative and elegant mechanism supported by structural data proposes that the phosphorylated C-terminus of LRP5/6 directly inhibits GSK-3 [95,96,97]. These two models are not mutually exclusive. Wnt signaling also promotes the mobilization of the Axin complex into multivesicular bodies, which may protect β-catenin from phosphorylation and degradation [98]. This sequestration mechanism occurs over several hours and does not explain the rapid reduction in GSK-3 enzymatic activity per se, whereas the dissociation of APC and the inhibition of GSK-3 occur within 30 min [80,86,89,90,91], but it may play an important role in maintaining active Wnt signaling over a more prolonged time. These mechanisms are non-exclusive, and all may contribute to the inhibition of GSK-3 and the stabilization of β-catenin by Wnts.

GSK-3 is also inhibited by RTK/AKT signaling, as first demonstrated for insulin-mediated signaling. AKT phosphorylates GSK-3α at serine-21 and Gsk-3β at serine-9, creating an intramolecular pseudosubstrate that mimics primed substrates and occludes the active site [51,60]. However, this inhibitory mechanism has no role in Wnt signaling [36,56,99]. GSK-3 bound to Axin is not phosphorylated by AKT and mice with the *Gsk3a^ser21ala^* and *Gsk3b^ser9ala^* double knockin, which cannot be phosphorylated by AKT, have no defects in Wnt signaling. Furthermore, Wnt signaling is rescued in *Gsk3* DKO mESCs by expression of *Gsk3b^ser9ala^*, which lacks the AKT phosphorylation site [36]. Similarly, Wnt signaling does not affect RTK/AKT signaling or cause phosphorylation of GSK-3 at serine-21/9. These findings demonstrate the insulation of the Wnt pool of GSK-3 from RTK/AKT signaling.

In summary, the canonical Wnt pathway signals through the Axin complex to inhibit GSK-3. For Wnt/β-catenin signaling, this prevents phosphorylation of β-catenin and thereby activates downstream Wnt/β-catenin target genes. However, this linear pathway to β-catenin is only part of the story.

### 3.2. When You Come to a Fork in the Road, Take It [100]: Divergence in Canonical Wnt Signaling

**Figure 2:**
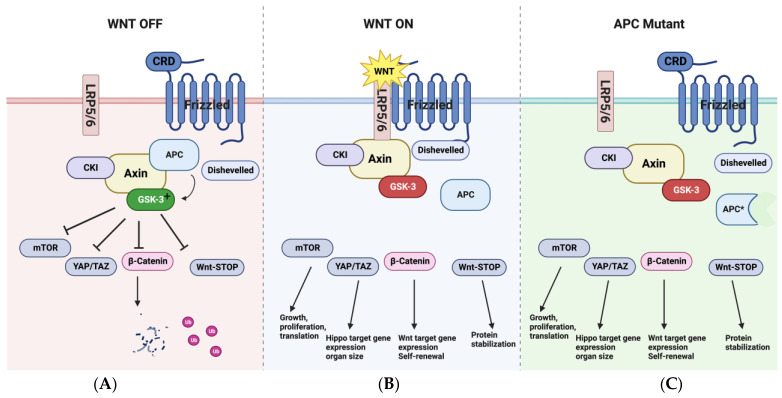
Multiple effectors of the Wnt pathway are regulated by APC, Axin, and GSK-3 independently of β-catenin. (**A**) In addition to phosphorylating β-catenin, the Axin complex, acting through GSK-3, suppresses mTORC1, targets multiple proteins for proteosomal degradation, and inhibits YAP/TAZ transcription factors (Hippo pathway). APC and GSK-3 also suppress ERK and BMP signaling and regulate mitotic spindle orientation (not shown in this figure). (**B**) Activation of the canonical Wnt pathway causes APC to dissociate from the Axin complex, reducing the catalytic activity of GSK-3. The phosphorylated C terminus of LRP5/6 also inhibits GSK-3 directly. GSK-3 inhibition activates mTORC1, YAP/TAZ, and the Wnt stabilization of protein (Wnt-STOP) pathway, in addition to β-catenin stabilization. (**C**) Oncogenic mutations in APC remove the domain required for Axin binding. As truncated APC does not bind to Axin, this mimics the dissociation of APC in response to Wnt signaling, thereby reducing GSK-3 activity and activating the downstream effectors β-catenin and mTORC1. For additional information on YAP/TAZ, see text. (Diagrams made with BioRender).

The Axin/APC/GSK-3 complex is a signaling hub with multiple effectors in addition to β-catenin (Figure 2) [6,7,94,101,102,103,104,105,106]. This was recognized early on from genetic studies in *C. elegans*, which showed that Wnt signaling through *Gsk3* is required for endoderm induction and mitotic spindle polarity independently of β-catenin [107]; the *APC*-like gene *apr* is also required for endoderm development [108], spindle orientation, and cell asymmetry [109] in *C. elegans.* Inhibition of GSK-3 or loss of function of *APC* also disrupts spindle orientation in mammalian cells, resulting in chromosomal misalignment [110,111,112].

An elegant biochemical analysis showed that Wnt signaling through the Axin/APC/GSK-3 complex activates the nutrient sensor mTOR complex 1 (mTORC1) in mammalian cells [103], as also observed in vivo in zebrafish embryos [94]. Inoki et al. showed that GSK-3 phosphorylates and enhances the activity of the tuberous sclerosis complex-2 protein (TSC2), an antagonist of mTORC1 activity. Wnt-dependent inhibition of GSK-3 through the APC/Axin complex impaired TSC2 function, resulting in the activation of mTORC1. GSK-3 phosphorylation promotes proteosomal degradation of multiple proteins [13,101,102], and Wnt-mediated inhibition of GSK-3 through APC and Axin stabilizes a subset of these proteins [13,101,102,106] through a mechanism termed “Wnt stabilization of proteins” or Wnt-STOP. Wnt signaling through APC also modulates the function of the Hippo pathway transcription factors YAP and TAZ in a manner that appears to be independent of β-catenin [113,114], although the nature of the interactions between Wnt and Hippo pathway components remains controversial, with somewhat distinct findings that are difficult to reconcile (discussed in detail below). Other parallels between APC and GSK-3 that are independent of β-catenin include modulation of retinoic acid synthesis [115,116], Bone Morphogenetic Protein (BMP) signaling [117,118,119], and Extracellular Signal-Regulated Kinase (ERK) signaling [120,121,122].

*Gsk3* knockdown/knockout or conditional *Apc* deletion in mice also causes expansion of hematopoietic stem and progenitor cells (HSCs) in bone marrow [123,124]. This expansion is transient and eventually leads to HSC depletion. HSPC expansion can be maintained by simultaneous inhibition of GSK-3 and mTORC1. Complete knockout of *Gsk3b* in hematopoietic cells causes a persistent myeloproliferative state with increased HSPCs/blast cells and marked expansion of mature granulocytes [125].

These are a few of the parallels between GSK-3 and APC in diverse organisms. Whether the effects of *APC* and *GSK3* loss of function are causally related and whether they are Wnt regulated has not been fully addressed in most cases, but the parallels are intriguing and are consistent with the hypothesis that APC is a positive regulator of GSK-3 in the Wnt pathway and that downstream effectors function independently of β-catenin. Thus, while β-catenin is the best characterized and most studied effector of canonical Wnt signaling, the pathway is more complex, with multiple potential effector pathways diverging from the APC/Axin/GSK-3 complex (Figure 2).

### 3.3. Wnt/GSK-3 Signaling in Cancer

*APC* is a classical tumor suppressor that was first identified because germline mutations cause the autosomal dominant cancer syndrome familial adenomatous polyposis (FAP), in which the loss of heterozygosity of *APC* causes hundreds of intestinal adenomas and inevitable colorectal cancer by the time patients reach their early 20s [126,127,128]. Somatic mutations in *APC* are also the most common early driver mutations in sporadic colorectal cancers; following the loss of *APC* and adenoma formation, additional somatic mutations, e.g., in *KRAS* or *TP53*, accumulate and lead to malignant transformation [127]. The importance of *APC* as a driver mutation was underscored by a study showing that restoring full-length *Apc* in mice with colon cancer initiated by oncogenic *Apc* mutations causes tumor regression despite the presence of *Kras* or *Tp53* mutations [129]. Human colon cancers that lack *APC* mutations frequently have activating mutations in *CTNNB1* (β-catenin) that prevent its phosphorylation by GSK-3, demonstrating a key epistatic relationship between *APC* and *CTNNB1*/β-catenin [130,131]. Activating mutations in *CTNNB1/*β-catenin (23–36%) and loss-of-function mutations in *AXIN* (5–10%) are common in hepatocellular cancer [132,133] and, along with other Wnt pathway mutations, are also observed in melanoma, uterine, gastric, and pancreatic ductal adenocarcinomas [133,134,135,136].

Given these data, it is widely accepted that *APC* mutations drive neoplasia by activating Wnt/β-catenin target genes, such as *MYC* (c-Myc) and *CCND1* (cyclin D1). However, the evidence for additional effectors of the Axin/APC/GSK-3 complex described above raises the possibility that Wnt-dependent, β-catenin-independent pathways contribute to the effects of *APC* loss in cancer. Pathogenic *APC* mutations tend to cluster in the mutation cluster region (mcr) and yield a truncated protein that does not bind to Axin. As APC is required in the Axin complex for maximal GSK-3 activity, oncogenic *APC* mutations reduce GSK-3 activity. Importantly, these mutations mimic physiological Wnt signaling, which drives transient dissociation of APC from the Axin complex [80,89,90,91], except that with truncating *APC* mutations, dissociation and activation persist indefinitely.

**Figure 3:**
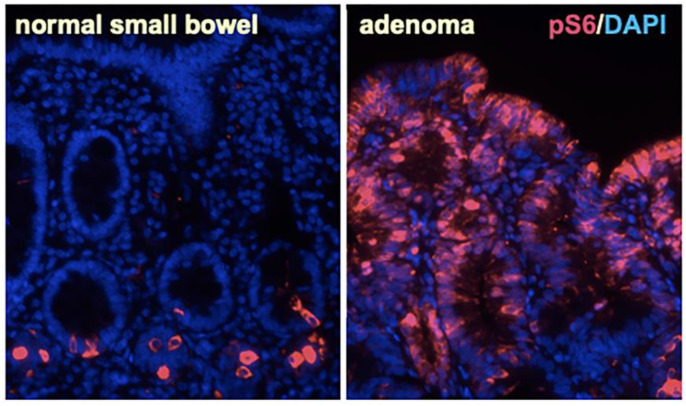
Activation of mTORC1 in intestinal adenomas from patients with FAP. Immunofluorescence for phosphorylated ribosomal protein S6 (pS6, magenta), a readout of mTORC1 activation, in biopsies of the small intestine from patients with familial adenomatous polyposis (FAP). Adenomas (**right**) were strongly positive for pS6 compared to adjacent normal intestine (**left**) in 8 of 9 patients examined. Nuclei are stained with DAPI (blue). Magnification 200×.

The reduced GSK-3 activity caused by oncogenic *APC* mutations stabilizes β-catenin but also activates mTORC1, YAP/TAZ (Hippo pathway), Wnt-dependent stabilization of proteins (Wnt-STOP), and other pathways. Activation of mTORC1 by *APC* truncations is clearly evident in zebrafish embryos with the homozygous *apc^mcr/mcr^* mutation [94], in adenomas from mice with the *Apc^min^* mutation [80], in sporadic colonic adenomas and colon cancer in humans [137,138,139], and in adenomas from patients with FAP (Figure 3). Consistent with a central role for mTORC1 in Apc-dependent tumorigenesis, Rapamycin blocks adenoma formation in *Apc^min^* mice [137,140,141,142,143,144,145,146,147,148]. Similarly, several phenotypes in *apc^mcr/mcr^* zebrafish embryos [149,150,151] caused by mTORC1 activation are reversed by the mTORC1 inhibitor Rapamycin and not by inhibition of β-catenin [94].

These data strongly support the notion that mTORC1 contributes to neoplasia caused by *APC* mutations, as also reported for many other cancers [139]. This does not conflict with the firmly established role of β-catenin in colonic neoplasia; indeed, *CTNNB1/*β-catenin overexpression in mouse colonic epithelial cells induces adenomas and *Ctnnb1* knockout blocks adenoma formation in *Apc* mutant mice [152,153,154,155]. Mutations in the GSK-3 phosphorylation site of β-catenin, which stabilize the protein and activate downstream signaling, also account for a substantial percentage of sporadic colon cancers that lack *APC* mutations and are the most common Wnt pathway mutation in HCC. But nuclear β-catenin is not frequently observed in early adenomas that form in FAP patients, sporadic human colonic polyps, or microadenomas from a rat FAP model, despite confirmed loss of heterozygosity of *APC* and increased cellular levels of β-catenin [156,157,158,159]. In this context, β-catenin could be active in the nucleus below the limit of detection by immunofluorescence, but it is also possible that elevated β-catenin caused by *APC* loss is insufficient to initiate hyperproliferation and microadenoma formation and that other pro-proliferative stimuli, including mTORC1 activation [80,94,105,139,142,143,144,145,146,160], could contribute to early adenoma formation.

The Hippo pathway regulates organ size, proliferation, and apoptosis by modulating levels of the transcription factors YAP and TAZ [161]. Multiple studies on interaction between Hippo and Wnt pathway components have been published, and while some of these findings are difficult to reconcile, several groups have shown that APC and GSK-3 suppress Hippo target gene expression by suppressing the level or function of YAP protein independently of β-catenin [113,162,163] (one group reported that β-catenin activates YAP transcription [164], which is distinct from but not incompatible with the other studies). Consistent with a suppressive role for APC and GSK-3, YAP and TAZ protein levels were increased by inhibition of GSK-3 or by oncogenic *APC* mutations. Although this regulation was mediated by APC and GSK-3 and independent of β-catenin, the specific, proposed mechanisms were distinct. Multiple groups also reported that *Yap* is genetically required for the development of adenomas driven by loss of *Apc*, as KO of *Yap* in Apc*^min^* mice abolished adenoma formation [113,163,165,166]. However, another paper presented data indicating that *Yap* and *Taz* suppress adenoma formation in the colon of mice with conditional deletion of Apc [167]. These seemingly opposing results are difficult to reconcile, and perhaps reflect the use of different experimental models of *Apc* loss of function and modulation of Hippo signaling. Nevertheless, apart from how YAP influences colonic epithelial regeneration and neoplasia, most studies are consistent with the direct regulation of Hippo signaling components by APC and GSK-3.

One might ask why *GSK3* mutations have not been identified as driver mutations in human cancers when loss of *GSK3* activates Wnt signaling similar to the loss of *APC* or *AXIN*. *GSK3A* and *GSK3B* are redundant in the Wnt pathway [36] and mostly redundant in other settings. As described above, three of the four *Gsk3* genes must be deleted in mouse ESCs to achieve modest activation of Wnt signaling and all four copies of *Gsk3* (*Gsk3a* and *Gsk3b)* must be deleted to achieve the high level of Wnt target gene activation observed with Apc mutations, making this an unlikely event. Furthermore, GSK-3 has multiple functions beyond suppression of Wnt signaling, and it is also possible that complete loss of *GSK3A/B* is not compatible with cancer cell viability, despite the clear vitality of *Gsk3a/b* DKO mESCs. The complexity of GSK-3 functions is likely a reason that GSK-3 inhibition can both enhance and impair oncogenic signaling, depending on context.

Most small-molecule GSK-3 inhibitors inhibit GSK-3α and GSK-3β equally. A reasonable concern for therapeutic applications of GSK-3 inhibitors is therefore that activation of Wnt/β-catenin signaling by GSK-3 inhibitors might lead to cancer. However, real-world data do not support this concern. Multiple large-scale, population-based retrospective studies of patients on the GSK-3 inhibitor lithium have shown no increased incidence of cancers [168,169] and in at least one study lithium was associated with reduced cancer risk [170]. Indeed, these findings have led to a phase 2 clinical trial to test the counterintuitive but intriguing hypothesis that low-dose lithium may prevent adenoma formation in FAP patients [171] (a similar approach was suggested by preclinical data in a B-cell lymphoma model, in which the inhibition of GSK-3 caused apoptosis by stabilizing and increasing levels of the c-Myc protein [172]). Therapeutic lithium does not achieve complete inhibition of GSK-3, so it remains possible that complete inhibition would still promote malignant transformation, but we are unaware of reports of malignancy induced by *Gsk3* DKO, which represents maximal inhibition of GSK-3. This conclusion is emphatically illustrated in a thorough review on the subject [16]. *Gsk3* DKO does enhance cell proliferation in some settings, however, including the developing CNS [173] and the adult hematopoietic system [123,125], and with other more potent transforming mutations, it could still, in principle, contribute to malignancy. The clinical data on lithium and the data on *Gsk3* DKOs summarized by Domoto et al. [16] are extensive, and from these many studies, the risk of cancer does not appear to be increased with GSK-3 inhibition, but caution is still needed as more potent GSK-3 inhibitors become available for clinical use.

## 4. Summary

The canonical Wnt pathway impinges on multiple effectors in addition to its well-established regulation of β-catenin and activation of Wnt target genes. The evidence supporting the role of β-catenin in development, tissue homeostasis, and disease is extensive and irrefutable but additional effectors of canonical Wnt signaling, including mTORC1, targeted protein degradation (Wnt-STOP), ERK, BMP, Hippo signaling, and likely others to be discovered, clearly play an important role in normal tissues and disease. A closer examination of these alternative effectors of the Wnt pathway revealed unexpected mechanisms for developmental phenotypes associated with *apc* mutations in zebrafish and may also identify new targets for cancer chemotherapy. GSK-3 functions at the bifurcation point of this pathway, directly modulating multiple downstream factors, and GSK-3 is also a core factor in RTK signaling and other pathways, a complexity of function that may explain why GSK-3 inhibition has been reported to have seemingly opposing effects depending on the tissue context. Despite the reasonable concern that GSK-3 inhibition could lead to malignancy, similar to mutations in *APC* or *Axin*, this has not yet been observed. This leaves open the opportunity for using small-molecule GSK-3 inhibitors for a number of illnesses including neurodegenerative and psychiatric disorders, as well as cancer.
